# A Workpeople's Gathering at Leicester

**Published:** 1914-05-02

**Authors:** 


### A Workpeople's Gathering at Leicester.

By the kindness of Sir Edward Wood, president of the Leicester Saturday Hospital Society, the delegates appointed by the contributing workpeople were on Saturday invited to inspect the new Children's Hospital, and to the number of 400 were subsequently entertained to tea in the grounds.

Mr. S. A. Gunson, one of the vice-presidents of the Society, was the chairman of the day, and hearty welcomes were given to the delegates by Mr. C. J. Bond, vice-president of the infirmary board, Mr. T. Fielding Johnson, jun., the chairman of the house committee, Mr. Harry Johnson (house governor), and others.

A resolution was passed deploring the indisposition of Sir Edward Wood, and a message sent conveying the best wishes of the delegates for his recovery.

Two pleasing announcements were made, the first that the workpeople of the Leicester Co-operative Society Boot and Shoe Branches had promised a piano for one of the new wards, and the second that Mr. J. I. Hallam, a life governor of the infirmary, had made an offer to the board to supply an electrical system of clocks to the new building.

